# Discovery of a Small Molecule Agonist of Phosphatidylinositol 3-Kinase p110α That Reactivates Latent HIV-1

**DOI:** 10.1371/journal.pone.0084964

**Published:** 2014-01-29

**Authors:** Geneviève Doyon, Michele D. Sobolewski, Kelly Huber, Deborah McMahon, John W. Mellors, Nicolas Sluis-Cremer

**Affiliations:** Division of Infectious Diseases, Department of Medicine, University of Pittsburgh School of Medicine, Pittsburgh, Pennsylvania, United States of America; Meharry Medical College, United States of America

## Abstract

Combination antiretroviral therapy (cART) can effectively suppress HIV-1 replication, but the latent viral reservoir in resting memory CD4^+^ T cells is impervious to cART and represents a major barrier to curing HIV-1 infection. Reactivation of latent HIV-1 represents a possible strategy for elimination of this reservoir. In this study we describe the discovery of 1,2,9,10-tetramethoxy-7H-dibenzo[de,g]quinolin-7-one (57704) which reactivates latent HIV-1 in several cell-line models of latency (J89GFP, U1 and ACH-2). 57704 also increased HIV-1 expression in 3 of 4 CD8^+^-depleted blood mononuclear cell preparations isolated from HIV-1-infected individuals on suppressive cART. In contrast, vorinostat increased HIV-1 expression in only 1 of the 4 donors tested. Importantly, 57704 does not induce global T cell activation. Mechanistic studies revealed that 57704 reactivates latent HIV-1 via the phosphatidylinositol 3-kinase (PI3K)/protein kinase B (Akt) signaling pathway. 57704 was found to be an agonist of PI3K with specificity to the p110α isoform, but not the p110β, δ or γ isoforms. Taken together, our work suggests that 57704 could serve as a scaffold for the development of more potent activators of latent HIV-1. Furthermore, it highlights the involvement of the PI3K/Akt pathway in the maintenance of HIV-1 latency.

## Introduction

Combination antiretroviral therapy (cART) can effectively suppress HIV-1 RNA levels to below 50 copies mL^−1^ in patient plasma. However, interruption of cART typically results in viremia rebound. Therefore, cART does not eliminate HIV-1 infection and residual low-level viremia has been detected using ultrasensitive assays in 80% of treated patients [1]–[4]. Latently infected resting memory CD4^+^ T cells are thought to constitute the major reservoir of HIV-1 persistence [5]–[8]. In this reservoir, the integrated provirus remains transcriptionally silent as long as the host cell is in a resting state [9]. Upon cellular activation, HIV-1 RNA is transcribed and virus is produced. The prevailing hypothesis in the field is that molecules that reactivate latent HIV-1 infection will purge this reservoir by inducing transcription of the latent provirus (i.e. the “kick”), thereby causing cells to undergo apoptosis (the “kill”) [10]. It should be noted, however, that in a recent study by Shan et al it was shown that after reversal of HIV-1 latency in an *in vitro* model, infected resting CD4+ T cells survived despite virl cytopathic effects, even in the presence of autologous cytolytic T lymphocytes from most patients on cART [26]. Despite this finding, several clinical trials are directly testing the “kick and kill” hypothesis using agents including the histone deacetylase (HDAC) inhibitor vorinostat (SAHA) [ClinicalTrials.gov Identifiers: NCT01319383 and NCT01365065] or disulfiram (DSF) [ClinicalTrials.gov Identifier: NCT0047732], a drug used to treat chronic alcoholism [11]. Encouragingly, recent data from the NCT01319383 trial showed that a single 400 mg dose of SAHA induced an increase in HIV-1 RNA expression in resting CD4^+^ T cells in 8 HIV-infected patients on suppressive cART [12].

While the “kick and kill” concept currently represents a promising avenue that could be scaled up for treatment of HIV-infected patients, the successful implementation of this approach is limited by the paucity of potent and safe inducers of latent HIV-1 gene expression. Importantly, many of these compounds either lack potency and specificity or have unfavorable toxicity profiles. For example, several studies have demonstrated that the latent HIV-1 reactivation activity of HDAC inhibitors is sub-optimal (compared to phytohaemagglutinin or anti-CD3/CD28 antibodies) and can vary considerably in resting T cells isolated from different HIV-1-infected donors [13], [14]. Additionally, HDAC inhibitors typically lack specificity and inhibit multiple HDAC isoforms (e.g. SAHA is a potent inhibitor of HDACs 1, 2, 3, 6 and 8) [16]. Similarly, the clinical utility of the PKC agonists prostrain and bryostatin may be limited by their unfavorable toxicity profiles. Prostratin induces global T cell activation [15], a phenotype that may induce clinical toxicity. In a recent phase II clinical trial for the treatment of ovarian cancer, bryostatin was found to cause severe myalgia in all study participants [17]. As such, there is an urgent need to develop potent and safe inducers of latent HIV-1 gene expression that could open new avenues to a finding a scalable and affordable cure for HIV-infected patients.

**Figure 1:**
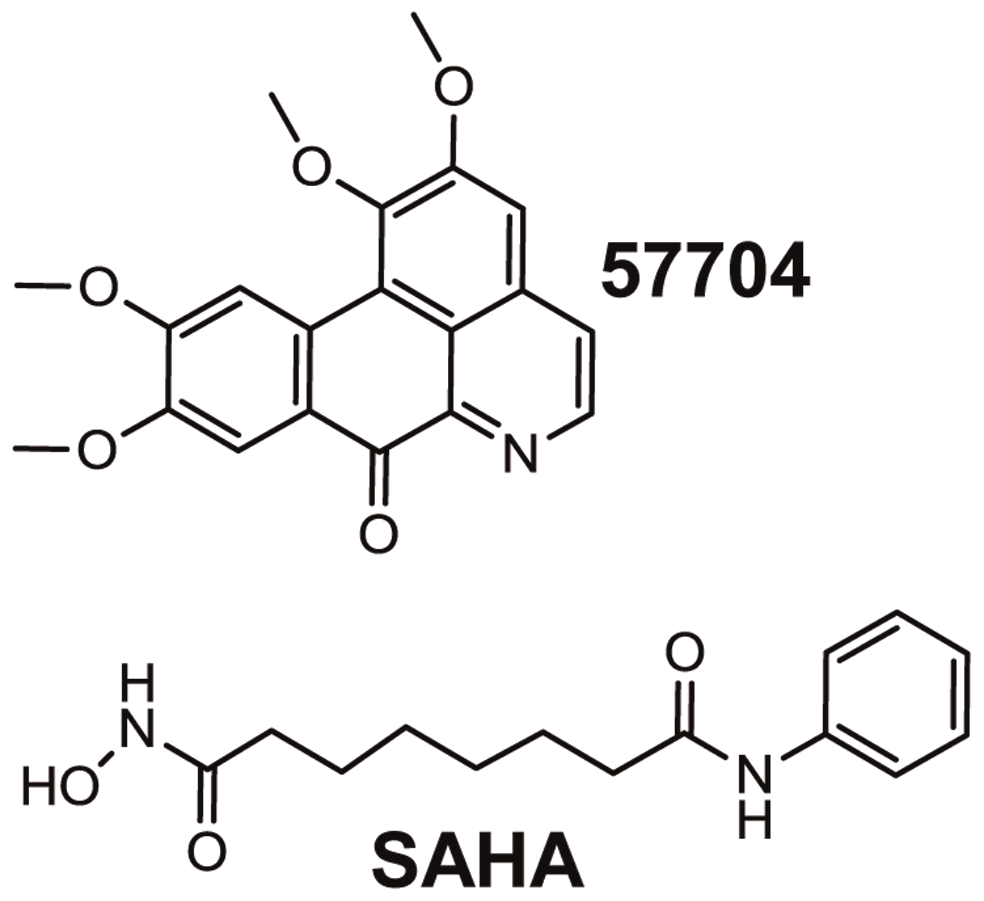
Chemical structures of 57704 and SAHA.

In this study, we described the discovery of 1,2,9,10-tetramethoxy-7H-dibenzo[de,g]quinolin-7-one (57704; Fig. 1). 57704 reactivates latent HIV-1 via the phosphatidylinositol 3-kinase (PI3K)/protein kinase B (Akt) signaling pathway through direct binding to, and activation of, the PI3K p110α isoform. 57704 could serve as a scaffold for the development of more potent activators of latent HIV-1.

## Materials and Methods

### Materials

The natural compound library was purchased from TimTec (Newark, DE). SAHA was from Enzo Life Sciences (Plymouth Meeting, PA). Wortmannin, SB203580, SP600125, Go6983, BIX-01294, cyclosporin A, DSF and prostratin were purchased from Sigma-Aldrich (St. Louis, MO). The PI3K isoform inhibitors were from BioVision (Milpitas, CA, USA). The Akt inhibitor IV and the NF-kB activation inhibitor were obtained from EMD Biosciences (Gibbstown, NJ). Akt and phospho-AKT were obtained from Cell Signaling Technology (Boston, MA). The β-actin antibody was obtained from Abcam (Cambridge, MA). The CD4-APC, CD8-PerCP, CD69-PE, CD38-PE and HLA-DR-PE antibodies were purchased from BD Biosciences (San Jose, CA). DNA oligonucleotide primers were synthesized by Integrated DNA Technologies (San Diego, CA). The recombinant purified HDAC isoforms were purchased from BPS Bioscience (San Diego, CA). Recombinant purified PI3K isoforms and the PI3K activity assay kit were purchased from Millipore (Billerica, MA). Cellular proteins were separated using 4–12% SDS-polyacrylamide gels from Invitrogen (Grand Island, NY). Immunodetection was performed using the Millipore SNAP i.d. ® (Billerica, MA).

### Cell line models of HIV latency

Three different cell lines of HIV-1 latency were used in this study. These included J89GFP, U1 and ACH-2 cells. The J89GFP cells were a kind gift from Dr. David Levy [18]. The ACH-2 and U1 cells were obtained through the AIDS Research and Reference Reagent Program, Division of AIDS, NIAID, NIH: Dr. Thomas Folks contributor [19]–[21]. Reactivation of latent HIV-1 in the J89GFP cells was measured by quantifying the percentage of EGFP positive cells using a BD FACScan flow cytometer with BD FACSDiva software (BD Biosciences, CA), as described previously. Reactivation of latent HIV-1 in the ACH-2 and U 1 cells was assessed by quantitative analysis of initiated and elongated HIV-1 transcripts, as described previously [16]. All 3 cell lines were maintained in RMPI 1640 medium supplemented with 10% FBS, 0.3 mg/mL L-glutamine, 100 U/mL penicillin, and 100 µg/mL streptomycin at 37°C in humidified air with 5% CO_2_.

### Cytotoxicity measurements

1×10^4^ cells were plated in 96-well plates with varying concentrations of 57704 or SAHA. Following a 24 hour incubation period, cell viability was measured using either the MTT (Roche Applied Science, Indianapolis, IN) or CellTiter 96 proliferation (Promega, WI) assay. The concentration of 57704 that decreased cell viability by 50% (i.e. 50% cytotoxic concentration; CC_50_) was calculated by regression analysis using SigmaPlot software.

### Blood donors

Healthy HIV-1 uninfected blood donors and HIV-1-infected donors on suppressive cART with plasma HIV-1 RNA less than 50 copies/mL for >2 year provided written informed consent and underwent a large volume phlebotomy (100–200 mLs). The protocol for blood donation was approved by the Institutional Review Board of the University of Pittsburgh (IRB #PRO10070203).

### Isolation of CD8+-depleted blood mononuclear cells (MNC) and quantitative assessments of HIV-1 RNA

CD8+T-cells were depleted from whole blood using RosetteSep Human CD8 Depletion Cocktail (StemCell Technologies, Vancouver, BC) according to manufacturer's instructions. 2×10^6^ CD4^+^ MNC cells were seeded in triplicate per treatment in 48-well plates and allowed to rest overnight. All wells were maintained in 81 nM efavirenz. Cells were treated with 57704 (1 or 5 µM), SAHA (0.5 or 2 µM), or vehicle control (0.1% DMSO). Activation controls were treated with CD3/CD28 Dynabeads (Life Technologies, Grand Island, NY). After 7 days of incubation, supernatants from triplicate wells were pooled, centrifuged (500 g×5 min), and stored at −80°C. HIV-1 RNA levels in supernatants were determined using the COBAS® AmpliPrep/COBAS® TaqMan® HIV-1 Test, v2.0 (Roche, Indianapolis, IN).

### Evaluation of global T cell activation

Peripheral blood MNC were obtained from the blood of healthy donors by Ficoll-Paque PLUS (GE Healthcare, Piscataway, NJ) density gradient centrifugation. T cells were isolated using the Pan T Cell Isolation kit (Miltenyi Biotec, Auburn, CA) according to manufacturer's instruction. Briefly, non-T cells were depleted from the MNC using biotin-conjugated antibodies to CD14, CD15, CD16, CD19, CD34, CD36, CD56, CD123, and CD235a and anti-biotin-labeled magnetic beads. Following exposure to 57704, SAHA or phytohaemagglutinin (PHA), the cells were washed and resuspended in 100 µl PBS that contained antibodies (αCD38-PE, αHLA-DR-FITC, or αCD69-PE) and incubated for 30 min. Cells were then washed twice with PBS, resuspended in PBS with 4% PFA and stored in the refrigerator until analysis. Samples were analyzed on a BD LSRII flow cytometer and analyzed using FACsDiva software (BD Biosciences, Mountain View, CA). Forward versus side scatter plot was used to define the live cell population and fluorescence gates were set based on isotype control fluorescence.

### PI3K activity assays

The production of phosphatidylinositol (3,4,5)-triphosphate (PIP3) by the four class I PI3K isoenzymes (p110 α, β, γ, δ) was assessed using the PI3 Kinase Activity/Inhibitor Assay Kit (Millipore, Billerica, MA) according to the manufacturer's instructions. This assay works on the principle that the PH domain of the protein GRP-1 binds PIP3 with high affinity and specificity. This protein binds to the glutathione plate and captures either the PIP3 generated as part of the kinase reaction or the biotinylated-PIP3 tracer included in the kit. The captured biotinylated-PIP3 is detected using streptavidin-HRP conjugate and a colorimetric read out (OD 450). Because the signal in this assay is inversely proportional to the kinase activity, the activity of 57704 was assessed in combination with the pan-PI3K inhibitor wortmannin.

### HDAC activity assays

The lysine deacetylase activity of HDACs 1, 2, 3, and 8 was assessed using the Fluorogenic HDAC Assay (BPS Bioscience, San Diego, CA) according to the manufacturer's instructions. The HDAC3 used in this assay was complexed with human nuclear receptor co-repressor 2 (NCOR2; amino acids 395–489), which is an activating co-factor of this HDAC isoform. All assays were carried out under steady-state conditions and the assay read-out was optimized for linearity both as a function of time and enzyme concentration. The formation of the fluorescent product was measured using a SpectraMax M2 Plate Reader (Molecular Devices, CA). The excitation and emission wavelengths were 360 nm and 450 nm, respectively. The concentrations of SAHA required to inhibit 50% of the deacetylase activity of an HDAC isoform (i.e. IC_50_) was calculated by regression analysis using SigmaPlot software (Systat Software, Inc., San Jose, CA).

## Results

### Discovery of 57704

**Figure 2:**
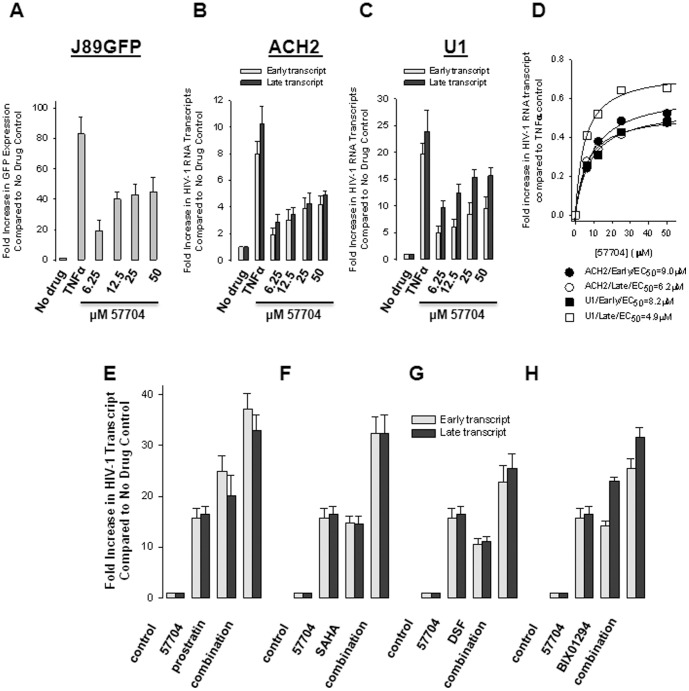
Latent HIV-1 reactivation activity of 57704, alone and in combination with other inducing agents, in the U1 and ACH2 cell line models of virus latency. HIV-1 expression was assessed by quantitative PCR analysis of early and late viral RNA transcripts. Where appropriate, TNFα was used as an activation control. (A) Latent HIV-1 reactivation of activity 57704 in the J89GFP cell line. An EC_50_ of 10.3±1.2 µM was determined for 57704 in this cell line. (B) Latent HIV-1 reactivation activity of 57704 in the ACH2 cell line. (C) Latent HIV-1 reactivation of activity 57704 in the U1 cell line. (D) Graph of activation of latent HIV-1 early and late gene transcript expression in the ACH2 and U1 cell lines. EC_50_ values were determined in Sigma Plot by nonlinear regression, as described previously. (E) Latent HIV-1 reactivation of 5 µM 57704 in combination with 1 µM prostratin in the U1 cell line. (F) Latent HIV-1 reactivation of 5 µM 57704 in combination with 5 µM SAHA in the U1 cell line. (G) Latent HIV-1 reactivation of 5 µM 57704 in combination with 2.5 µM DSF in the U1 cell line. (H) Latent HIV-1 reactivation of 5 µM 57704 in combination with 20 µM BIX-01294 in the U1 cell line. Data represent the mean ± standard deviation from 3 replicate experiments.

To identify novel activators of latent HIV-1 expression, we screened 640 pure natural products from the TimTec Natural Compound Library at a concentration of 10 ng/mL for 24 hrs in a Jurkat cell (J89GFP) model of latency for their cytotoxicity and ability to reactivate HIV-1 expression. J89GFP cells contain a stably integrated, full-length HIV-1 provirus with an enhanced GFP (EGFP) reporter incorporated into the viral genome [18]. The viral genome in these cells is transcriptionally silent. However, upon stimulation viral transcription is activated and viral expression can be measured by EGFP production. Seven compounds (1.1%) were identified that reactivated latent HIV-1 expression, none of which showed significant cytotoxicity at the single concentration tested (data not shown). Following extensive analyses in different cell line models of HIV-1 latency we found that 57704 (Fig. 1) was one of the more robust analogs identified in the screen. The effective concentration of compound that reactivated 50% of latent HIV-1 gene expression (i.e. EC_50_) ranged from ∼5 to 9 µM in the ACH2 and U1 cell lines of virus latency (Fig. 2B, C, D). By comparison, in J89GFP cells the EC_50_ was calculated to be 10.3±1.2 µM (Fig. 2A). The EC_50_ values for SAHA ranged from ∼1 to 5 µM in the same cell lines (data not shown). Importantly, 57704 was significantly less cytotoxic than SAHA in several different cell lines and in CD8+-depleted MNC (Table 1). Of note, the latent HIV-1 reactivation activity of 57704 was increased in an additive manner when combined with other inducing agents including prostratin (Fig. 2E), SAHA (Fig. 2F), DSF (Fig. 2G) and the histone methyltransferase inhibitor BIX-01294 (Fig. 2H).

**Table 1:** Cytotoxicity of 57704 and SAHA in different cells.

Compound	Jurkat^1^ CC_50_ (µM)	HeLa^1^ CC_50_ (µM)	293T^1^ CC_50_ (µM)	CD8+-depleted MNC^2^ CC_50_ (µM)
57704	92.9±7.5	293.2±56.42	105.5±31.4	80
SAHA	10.0±0.1	11.3±0.3	14.1±0.2	0.7

1 Data represent the mean ± standard deviation from 3 replicate experiments.

2 Data represent the mean from 2 independent replicate experiments.

### 57704 increases HIV-1 expression in CD8+-depleted MNC from HIV-1-infected donors on suppressive cART without inducing global T cell activation

**Figure 3:**
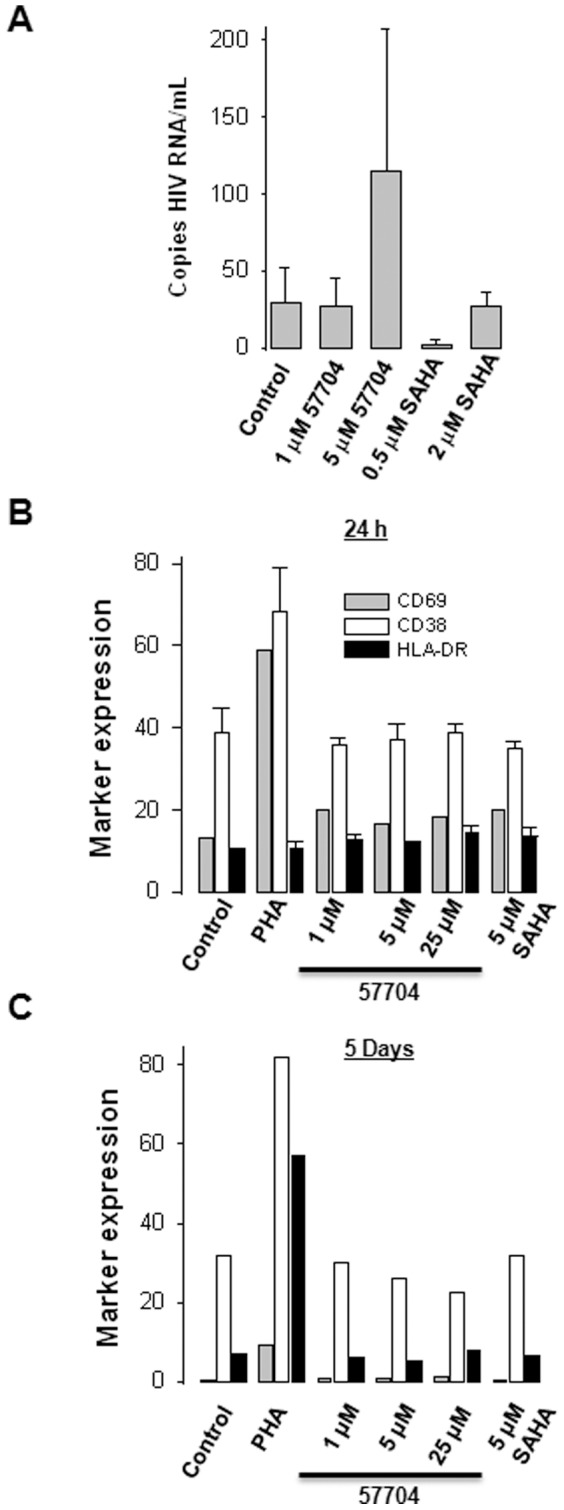
Latent HIV-1 reactivation activity of 57704 and T cell activation in CD8+-depleted MNC. (A) Quantitation of cell-free HIV-1 RNA in the culture supernatants of CD8+-depleted blood MNC incubated with 57704 or SAHA. (B) Surface expression of CD69, CD38 and HLA-DR after 24 h in purified T cells following exposure to 10 µg/mL PHA, 57704 or SAHA. (C) Surface expression of CD69, CD38 and HLA-DR after 5 days in purified T cells following exposure to 10 µg/mL PHA, 57704 or SAHA.

We compared the activity of 57704 to reactivate latent HIV-1 from CD8+-depleted blood MNC from 4 different cART-treated individuals with plasma HIV-1 RNA<50 copies/mL (Table 2, Fig. 3A). SAHA was included as a control. 5 µM 57704 increased HIV-1 RNA in culture supernatants ∼4-fold compared to the control (Fig. 3A) although significant variability was observed between donors (Table 2). Specifically, increases in HIV-1 RNA levels in culture supernatant compared to the vehicle control were detected in the CD8^+^-depleted blood MNC from donors 1 and 2 but not in donors 3 and 4. Although the mean supernatant HIV-1 RNA copy number determined for the 4 donors after treatment with 1 µM 57704 was comparable to that of controls, higher levels were detected in donors 3 and 4. By contrast, SAHA exhibited a very limited capacity to increase HIV-1 expression in the CD8+-depleted MNC across all four donors (Table 2). Since agents that induce latent HIV-1 through global T cell activation are toxic, we next assessed the 57704's capacity to induce this phenotype. Purified T cells were treated with 10 µg/mL PHA (positive control), 5 µM SAHA or different concentrations of 57704 for up to 5 days, and then stained for surface expression of CD69, CD38 and HLA-DR (Fig. 3B, C). 57704 did not up-regulate the surface expression of any of these markers, even after 5 days of exposure to drug (Fig. 3C). Taken together, these data indicate that 57704 increases HIV-1 expression in CD8+-depleted MNC from HIV-1-infected donors without inducing global T cell activation.

**Table 2:** Latent HIV-1 reactivation activity of 57704 in CD8+-depleted MNC from HIV-1-infected donors on suppressive cART.

Donor	CD4+ Count	Viral Load (RNA/mL)	ART Regimen	Copies HIV RNA/mL control	Copies HIV RNA/mL 1 µM 57704	Copies HIV RNA/mL 5 µM 57704	Copies HIV RNA/mL 0.5 µM SAHA	Copies HIV RNA/mL 2 µM SAHA
1	484	not detected	Reyataz, Epzicom, Norvir	96	0	389	0	51
2	777	<40	Reyataz, Truvada, Norvir	0	79	35	0	24
3	713	<20	Reyataz, Truvada, Norvir	0	10	0	10	10
4	571	not detected	Atripla	20	20	27	0	23

### Mechanism of action studies

**Figure 4:**
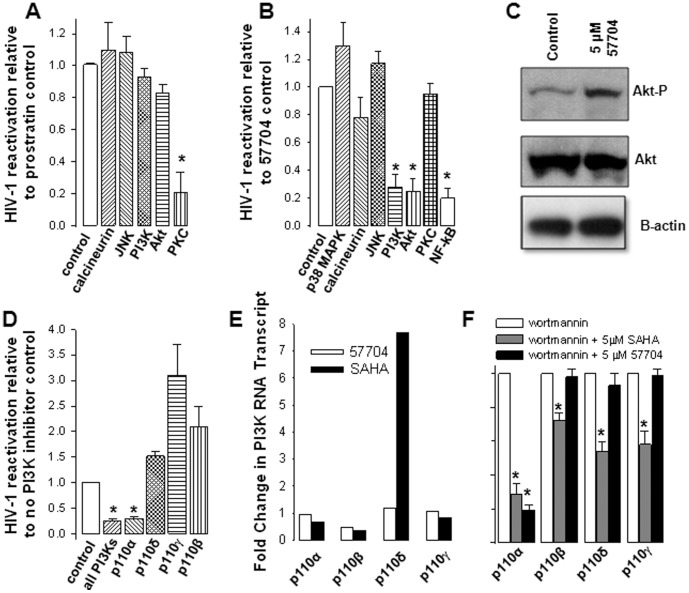
57704 reactivates latent HIV-1 via the PI3K/Akt signaling pathway. (A) Latent HIV-1 reactivation activity of prostratin in the absence (control) or presence of inhibitors of calcineurin (1 µM cyclosporin A), JNK (1 µM SP00125), PI3K (20 nM wortmannin), Akt (500 nM Akt Inhibitor IV) and PKC (500 nM Go6983). (B) Latent HIV-1 reactivation activity of 57704 in the absence (control) or presence of inhibitors of p38 MAPK (50 nM SB203580), calcineurin, JNK, PI3K, Akt, PKC and NF-κB (20 µM NF- κB activation inhibitor). (C) Western blot analysis of phosphorylated Akt in J89GFP treated with 5 µM 57704. (D) Latent HIV-1 reactivation activity of 57704 in the absence (control) or presence of a pan-inhibitor of PI3K (15 µM LY294002) or inhibitors targeting the p110α (2 µM PI-103), p110δ (5 µM IC87114), p110γ (2 µM AS-605240) or p110β (2 µM TGX-221) isoforms. (E) PI3K isoform transcript levels in cells treated with 5 µM 57704 or 5 µM 57704 SAHA.(F) Inhibition of the PI3K p110α, β, δ and γ isoforms by 100 nM wortmannin alone or in combination with 5 µM 57704 or 5 µM 57704 SAHA.

To gain insight into the mechanism by which 57704 reactivates latent HIV-1, we evaluated its activity in combination with inhibitors of different signaling pathways or kinases in J89GFP cells (Fig. 4A, B). We found that the activity of 57704 was significantly attenuated by the PI3K inhibitor wortmannin, by the Akt Inhibitor IV, and by the NF-κB inhibitor 6-amino-4-(4-phenoxyphenylethylamino) quinazoline. By contrast, its activity was unaffected by the PKC inhibitor Gö6983, by the c-Jun N-terminal kinase (JNK) kinase inhibitor SP600125, by the p38 mitogen-activated protein kinase inhibitor SB203580, and by the NFAT inhibitor cyclosporin A. Consistent with its mechanism of action, the latent HIV-1 reactivation activity of prostratin was attenuated when combined with the PKC inhibitor Gö6983, but remained unaffected by inhibitors of calcineurin, JNK, PI3K and Akt (Fig. 4A). Taken together, these data suggested that 57704 reactivated latent HIV-1 via the PI3K/Akt signaling pathway. To determine whether 57704 directly activated the PI3K/Akt signaling pathway, we next assessed the levels of Akt phosphorylation in the J89GFP cells after exposure to 5 µM 57704. Western blot analysis clearly demonstrated that 57704 increased Akt phosphorylation (Fig. 4C).

The class I PI3Ks are heterodimeric enzymes formed by association of a p110 catalytic subunit with a p85 regulatory subunit. There are 4 variants of the p110 catalytic subunit designated p110α, β, δ or γ [22]. To determine whether 57704 displayed PI3K isoform specificity, we first assessed its activity in J89GFP cells in combination with inhibitors specific to each PI3K isoform (Fig. 4D). We found that 57704's activity was significantly attenuated by the pan PI3K inhibitor LY 294002 and by the PI3K p110α specific inhibitor PI-103. By contrast, its activity was not reduced by inhibitors of PI3K p110β, p110δ or p110γ. Of note, AS-605240 and TGX-221 which target PI3K p110γ and p110β, respectively, moderately enhanced the latent HIV-1 reactivation activity of 57704. To determine whether 57704 directly impacted the gene expression levels of the PI3K isoforms, we assessed RNA transcript levels in J89GFP cells treated with either 57704 or SAHA (Fig. 4E). We found that 57704 moderately decreased PI3K p110β RNA levels (∼2-fold) but had no effect on p110α, p110δ or p110γ. SAHA also moderately decreased PI3K p110β RNA levels (∼2-fold), but was found to significantly up-regulate p110δ gene expression. We next assessed 57704's activity against recombinant purified PI3K isoforms using an assay developed by Millipore. Briefly, in this assay the PIP3 generated as part of the kinase reaction or the biotinylated-PIP3 tracer included in the kit is captured on a glutathione plate via its interaction with the pleckstrin homology (PH) domain of the glutathione S-transferase (GST)-fusion protein general receptor for phosphoinositides-1 (GRP-1). The captured biotinylated-PIP3 is then detected using a streptavidin-HRP conjugate and a colorimetric read out. Because the signal of the assay is inversely proportional to PI3K activity, we assessed the activity of 57704 in combination with wortmannin. We found that 57704 acted as an agonist in that it abrogated the ability of wortmannin to inhibit PI3K p110α (Fig. 4E,F) but not PI3K p100β, δ or γ (Fig. 4F). Prior studies demonstrated that SAHA and other HDAC inhibitors also activated the PI3K/Akt signaling pathway [16], [23]. Therefore, we also assessed the ability of SAHA to impact the activity of PI3K. Interestingly, SAHA also acted as an agonist of PI3K p110α. (Fig. 4F) However, it also activated the PI3K p100β, δ and γ isoforms (Fig. 4F). In light of this finding, we next assessed whether 57704 inhibited the activity of the class I HDAC isoforms, HDAC1, HDAC2, HDAC3 and HDAC8, using recombinant purified enzymes (Table 3). SAHA was included as a control in these experiments. 57704 exhibited no discernable inhibitory activity toward any of the HDAC isoforms (Table 3).

**Table 3:** In vitro activity of 57704 and SAHA against class I HDAC isoforms.

HDAC Isoform	SAHA IC_50_ (nM)	57704 IC_50_ (µM)
HDAC1	13.7±0.15	>50
HDAC2	62.0±0.15	>50
HDAC3	869±0.15	>50
HDAC8	6.8±0.15	>50

Data represent the mean ± standard deviation from 3 replicate experiments.

## Discussion

The latent reservoir in resting memory CD4+ T cells represents a major obstacle towards curing HIV-1 infection. The mechanisms that lead to HIV-1 latency in CD4+ T cells are still not completely understood. Indeed, there is ample evidence that multiple restrictions prevent the emergence of virus from latency, including both cellular and viral factors [24]. Therefore, a better understanding of the establishment and maintenance of HIV-1 latency and identification of pharmacologic targets will open avenues to rational therapeutic approaches for clearing infection.

In this study, we show that 57704 – a novel agonist of PI3K p110α – can reactivate latent HIV-1 in different cell lines of virus latency and increase HIV-1 expression in CD8+-depleted blood MNC from infected individuals on suppressive cART. In this regard, its activity in CD8+-depleted MNC was more robust than that of SAHA, which is currently being tested in clinical trials for its potential to reduce or eliminate the latent HIV-1 reservoir. Importantly, 57704 was found to reactivate latent HIV-1 without inducing global T cell activation. As such, 57704 serves as the prototype of a new mechanistic class of agents that could help purge the latent HIV-1 reservoir. Furthermore, our data validate PI3K p110α as a target for the discovery of more potent activators of latent HIV-1 expression.

The PI3K/Akt signaling pathway appears to play a key role in the maintenance of HIV-1 latency. Indeed, previous studies have demonstrated that hexamethylene bisacetamide (HMBA) transiently activates this pathway, which leads to phosphorylation of HEXIM1 and the subsequent release of active positive transcription elongation factor b (P-TEFb) from its transcriptionally inactive complex with HEXIM1 and 7S small nuclear RNA (snRNA). As a result, P-TEFb is recruited to the HIV-1 promoter to stimulate transcription elongation and viral production [25]. Similarly, SAHA and other HDAC inhibitors have been shown to activate this pathway in several subpopulations of T cells, including memory T cells [16], [23]. Importantly, inhibition of PI3K significantly reduces the latent reactivation activity of HDAC inhibitors [16], [23]. The mechanism by which HMBA activates the PI3K/Akt pathway is not known. However, in this study we show that SAHA, like 57704, acts as an agonist of PI3K p110α. However, unlike 57704, SAHA also shows agonistic activity toward the other PI3K isoforms. Therefore, 57704 and SAHA activate the PI3K/Akt signaling pathway via a similar mechanism of action. However, it should be noted that 57704 is not an HDAC inhibitor.

The class I PI3Ks include four isoforms consisting of two subdivisions, namely, class IA (PI3K p110α, β and δ) activated by tyrosine kinases, antigen and cytokine receptors, and class IB (PI3K p110γ) activated by G-protein-coupled receptors [22]. The class IA isoforms share 42–58% amino acid sequence. PI3K p110γ shares 36% identity with p110α. Despite largely conserved active sites (they all catalyze the same reaction), isoform specific inhibitors have been developed to each of the PI3K isoforms [22]. Interestingly, we found that 57704 shows unique specificity to PI3K p110α. Its interaction with this isoform and the mechanism by which it enhances kinase activity are not known, but warrant further investigation. Similarly, the contribution of each PI3K isoform in the establishment and maintenance of HIV-1 latency is not known. In this regard, additional research is required to determine whether an agonist with broader specificity to other PI3K isoforms may elicit a more pronounced effect in reactivating latent HIV-1 expression.

In conclusion, we have discovered that 57704, a novel small molecule agonist of PI3K p110α, can reactivate latent HIV-1. The 57704 pharmacophore could serve as a scaffold for the development of more potent activators of latent HIV-1 that could be used, possibly in combination with other agents, to induce expression of the latent reservoir in HIV-1-infected individuals.
